# Isolation and Identification of Cytotoxic Compounds from *Aeschynomene fascicularis*, a Mayan Medicinal Plant

**DOI:** 10.3390/molecules200813563

**Published:** 2015-07-24

**Authors:** Edgar E. Caamal-Fuentes, Sergio R. Peraza-Sánchez, Luis W. Torres-Tapia, Rosa E. Moo-Puc

**Affiliations:** 1 Unidad de Biotecnología, Centro de Investigación Científica de Yucatán (CICY), Calle 43 No. 130, Col. Chuburná de Hidalgo, Mérida, Yucatán 97200, Mexico; E-Mails: ecaamal@gmail.com (E.E.C.-F.); speraza@cicy.mx (S.R.P.-S.); lwtorres@cicy.mx (L.W.T.-T.); 2 Unidad de Investigación Médica Yucatán, Unidad Médica de Alta Especialidad, Centro Médico Ignacio García Téllez, Instituto Mexicano del Seguro Social (IMSS), Calle 41 No. 439, Col. Industrial, Mérida, Yucatán 97150, Mexico

**Keywords:** Fabaceae, flavonoids, Mayan medicinal plant, cytotoxic activity, antiproliferative activity

## Abstract

The plant *Aeschynomene fascicularis* (Fabaceae) has been used in Mayan traditional medicine in the Yucatan peninsula. However, the compounds present in the plant responsible for its curative properties have not yet been investigated. *Aeschynomene fascicularis* root bark was extracted with 100% methanol to obtain a crude extract. The methanol extract was partitioned successively with solvents with increasing polarity to obtain the corresponding hexane (Hx), dichloromethane (DCM) and ethyl acetate fractions (EtOAc), as well as a residual water-alcoholic fraction. These fractions were tested for their cytotoxic activities using an MTT assay against Hep-2 cancer cell lines. The Hx fraction led to the isolation of spinochalcone C (**1**), spinochalcone A (**2**), isocordoin (**3**) and secundiflorol G (**4**). Their structures were identified based on spectroscopic evidence and chemical properties. All compounds were subjected to cytotoxicity and antiproliferative assays against a panel of seven cell lines, including one normal-type cell line. Spinochalcone A (**2**) exhibited cytotoxic activity against DU-145 cell line and antiproliferative activity against the KB cell line. Secundiflorol G (**4**) showed strong cytotoxic activity towards KB and Hep-2 cell lines. In addition, isocordoin (**3**) showed moderate activity on KB, Hep-2 and DU-145 cell lines. The active Compounds **2**, **3** and **4** are potential therapeutic entities against cancer.

## 1. Introduction

The flora of the Yucatan peninsula is rich in vascular plants, including 2600–3000 species [1]. Some of these species have been used in traditional medicine by local communities for the treatment of a large number of diseases [2,3,4]. Therefore, based on their traditional uses, these plants constitute good sources of new molecules that can be used for the treatment of different ailments. Furthermore, 50% of anticancer drugs used in clinical trials have been isolated from plants [5]. Consequently, traditional medicinal plants can serve as potential sources in the development of new, more effective anticancer agents for future therapy.

In the Yucatan, medicinal plants have been used in urban and rural communities as a common practice for the control of many types of diseases, including cancer. Moreover, they are used in the treatment of conditions consistent with the symptoms of cancer: abscesses, calluses, corns, hard lumps, polyps, tumors or warts. In a preliminary screening for cytotoxic constituents from plants used in Mayan traditional medicine to treat cancer-like symptoms, 21 species were collected, and the crude methanol extracts of various plant parts were subjected to a cytotoxicity assay against a panel of cancer cell lines [6]. The results indicated that *Aeschynomene fascicularis* root bark has a pronounced cytotoxic activity on cancer cell lines.

*Aeschynomene fascicularis* Cham. and Schltdl. (Fabaceae) is a shrub extensively distributed from Mexico to Colombia. This plant is commonly known as *kabal pich* by the ancient inhabitants of the Yucatan Peninsula, and it has been used in Mayan traditional medicine to treat superficial tumors [2]. Few chemical studies on species of *Aeschynomene* genus have been done [7]. Recently, our group has isolated pterocarpans from the methanol extract of *A. fascicularis* [8]. Therefore, there are no studies yet on the cytotoxic active principles of the medicinal plant *A. fascicularis*.

Continuing our effort to search for novel anti-cancer agents from Mayan medicinal plants of the Yucatan peninsula, we performed a guided fractionation of the root bark extract to isolate and identify the cytotoxic compound(s) from this plant. Thus, the aim of this study was to isolate and identify the active cytotoxic compounds from *A. fascicularis*.

## 2. Results and Discussion

### 2.1. Identification of Compounds **1**–**4**

The active hexane fraction obtained from the methanol extract of *A. fascicularis* root bark was subjected to chromatographic purification, resulting in the isolation of four compounds. The chemical structures of all compounds were determined by the analysis of their NMR spectra (^1^H, ^13^C, HSQC and HMBC) and mass spectra and by comparison of these data with those published in the literature.

**Figure 1:**
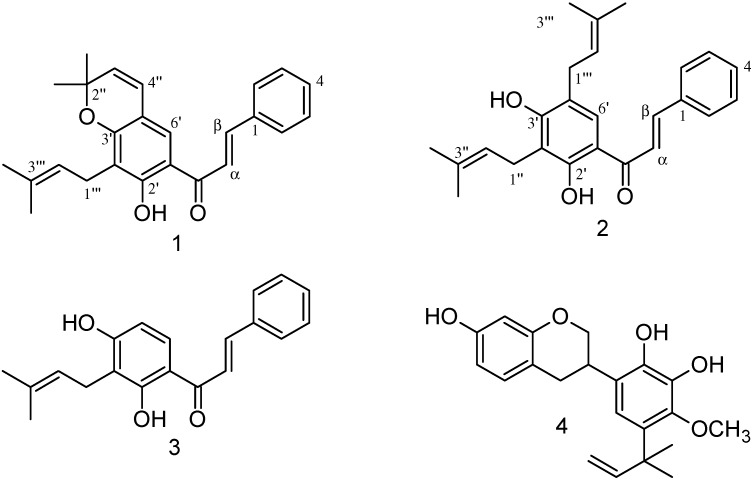
Chemical structures from flavonoids isolated from *Aeschynomene fascicularis* **1**–**4**.

Compound **1**: yellow oil. UV λ_max_ (CHCl_3_) nm: 239, 308; FTIR (film) ν_max_ cm^−1^: 3385, 1649, 1600, 1450, 1332, 1122; EI-MS 70 eV, *m*/*z* (relative intensity %): 374 ([M]^+^, 30), 359 (100), 303 (25), 255 (20), 131 (15), 103 (17). For ^1^H-NMR (400 MHz, CDCl_3_) and ^13^C-NMR (100 MHz, CDCl_3_) data, see Table 1. Compound **1** showed a molecular mass of 374 a.m.u. Its FTIR spectrum showed bands at 3385 and 1649 cm^−1^, which in conjunction with the ^1^H-NMR signal of a chelated proton at δ 13.74 (1H, s), signals due to two *trans*-olefinic protons at δ 7.57 (1H, d, *J* = 15.4 Hz) and δ 7.86 (1H, d, *J* = 15.4 Hz), and an α,β-unsaturated carbonyl signal at δ 191.9 in the ^13^C-NMR spectrum, confirmed the structure of a chalcone. Additionally, the ^1^H-NMR spectrum showed signals in the aromatic region belonging to a singlet at δ 7.40 and two sets of multiplets at δ 7.43 (3H) and δ 7.65 (2H) characteristic of protons in penta-substituted and mono-substituted rings, respectively. The presence of two *cis*-olefinic protons at δ 5.59 (1H, d, *J* = 9.8 Hz) and δ 6.32 (1H, d, *J* = 9.8 Hz) along with two signals for two methyls (δ 25.9 and 18.0) and a signal at δ 77.8 due to a quaternary carbon in the ^13^C-NMR spectrum are consistent with the occurrence of a 2,2-dimethyl-pyran ring. Furthermore, signals at δ 1.46 (6H), δ 3.36 (1H, d, *J* = 7.3 Hz) and a multiplet at δ 5.26 (1H) indicate the presence of an isoprenyl group. The position of all protons and carbons was established by combining the two-dimensional HSQC and HMBC experiments. The assigned structure (Figure 1) corresponds to spinochalcone C, previously reported from *Tephrosia spinosa* [9].

**Table 1:** NMR spectra of compounds **1** and **2**.

Position	Spinochalcone C (1)		Spinochalcone A (2)	
	δ_H_ (*J* = Hz)	δ_C_	δ_H_ (*J* = Hz)	δ_C_
1		135.0		135.2
2	7.65 m	128.6	7.62 m	128.5
3	7.43 m	129.0	7.40 m	129.0
4	7.43 m	130.6	7.40 m	130.6
5	7.43 m	129.0	7.40 m	129.0
6	7.65 m	128.6	7.62 m	128.5
A	7.57 d (15.4)	120.6	7.56 d (15.4)	120.7
B	7.86 d (15.4)	144.0	7.86 d (15.4)	144.0
1′		113.8		113.7
2′		164.4		162.6
3′		117.1		114.5
4′		158.0		160.4
5′		113.2		119.0
6′	7.40 s	125.2	7.55 s	128.6
1′′			3.46 d (7.0)	22.0
2′′		77.8	5.30 m	121.4
2′′-(CH_3_)_2_	1.46 s	28.6		
3′′	5.59 d (9.8)	128.7		135.0
3′′-(CH_3_)_2_			1.76 s	25.9
			1.84 s	18.0
4′′	6.32 d (9.8)	121.7		
1′′′	3.36 d (7.3)	21.6	3.33 d (7.0)	29.3
2′′′	5.26 m	122.0	5.30 m	122.0
3′′′		131.7		134.8
3′′′-(CH_3_)_2_	1.69 s	25.9	1.79 s	25.9
	1.82 s	18.0	1.80 s	18.0
2′-OH	13.74 s		13.63 s	
4′-OH			6.31 s	
C=O		191.9		192.1

Compound **2**: yellow amorphous solid. UV λ_max_ (CHCl_3_) nm: 238, 322; FTIR (film) ν_max_ cm^−1^: 3390, 1634, 1608, 1567, 1362, 1147; ^1^H- (400 MHz, CDCl_3_) and ^13^C- (100 MHz, CDCl_3_) NMR: see Table 1; EI-MS 70 eV, *m*/*z* (relative intensity %): 376 ([M]^+^, 100), 333 (40), 305 (45), 265 (30), 201 (40), 161 (60), 131 (40), 103 (35). Compound **2** has a molecular mass of 376 a.m.u. A set of signals in the ^1^H, ^13^C-NMR and FTIR spectra indicated the presence of a chalcone skeleton similar to Compound **1**. However, **2** presents two isoprenyl groups confirmed by the presence of methyl signals at δ 1.76, 1.79, 1.80 and 1.84 (3H each), two signals at δ 3.33 and 3.46 (2H, *J* = 7.0 Hz) and a multiplet at δ 5.30 (2H). The location of all protons and carbons was established by combining the two-dimensional HSQC and HMBC experiments. The assigned structure (Figure 1) corresponds to spinochalcone A, previously reported from *Tephrosia spinosa* [10]. Complete NMR assignments of **1** and **2** are reported here for the first time.

Compound **3**: yellow amorphous solid. FTIR (film) ν_max_ cm^−1^: 3231, 1629, 1613, 1480, 1444, 1362, 1234; EI-MS 70 eV, *m*/*z* (relative intensity %): 308 ([M]^+^, 55), 265 (90), 231 (12), 149 (100), 131 (37), 103 (40), 77 (20). ^1^H-NMR (400 MHz, CDCl_3_): δ 1.78 (3H, s, 3′′-Me), 1.85 (3H, s, 3′′-Me), 3.49 (2H, d, *J* = 7.0 Hz, H-1′′), 5.31 (1H, t, *J* = 7.0 Hz, H-2′′), 6.44 (1H, d, *J* = 8.9 Hz, H-5′), 7.43 (5H, m, B ring protons), 7.60 (1H, d, *J* = 15.4 Hz, H-α), 7.74 (1H, d, *J* = 8.9 Hz, H-6′), 7.89 (1H, d, *J* = 15.4 Hz, H-β), 13.76 (1H, s, OH). ^13^C-NMR (100 MHz, CDCl_3_): δ 18.2 (3′′-Me), 22.0 (C-1′′), 26.0 (3′′-Me), 108.1 (C-5′), 114.3 (C-1′), 114.4 (C-3′), 120.8 (C-α), 121.3 (C-2′′), 128.7 (C-2/C-6), 129.2 (C-3/C-5), 129.6 (C-6′), 130.8 (C-4), 135.1 (C-1) 136.1 (C-3′′), 144.5 (C-β), 162.0 (C-4′), 164.2 (C-2′), 192.3 (C=O). Compound **3** gave identical spectral features with those described for isocordoin, which has previously been isolated from *Cordoa piaca* [11] and from the root of *Lonchocarpus xuul* [12].

Compound **4**: brown-red amorphous solid. ${\left[\alpha \right]}_{D}^{20}$ +9.5 (*c* 0.02, CHCl_3_); UV λ_max_ (CHCl_3_) nm: 238, 283; FTIR (film) ν_max_ cm^−1^: 3385, 1618, 1598, 1506, 1454, 1275, 1157, 1116. In the ^1^H-NMR spectrum, a set of mutually-coupled five protons (δ 2.88 (1H, dd, *J* = 16.0, 4.0 Hz), 3.02 (1H, ddd, *J* = 16.0, 10.0, 3.0 Hz), 3.52 (1H, m), 4.09 (1H, t, *J* = 10.0 Hz) and 4.36 (1H, d, *J* = 10.0 Hz)) suggested the presence of an isoflavan skeleton. The ^1^H-NMR spectrum further exhibited the presence of a methoxyl at δ 3.74 (s), and signals of a typical α,α-dimethyl-allyl group were observed (δ 1.37 (3H, s), 1.51 (3H, s), 4.97 (1H, dd, *J* = 18.0, 1.0 Hz), 5.02 (1H, dd, *J* = 11.0, 1.0 Hz), and 6.13 (1H, ddd, *J* = 18.0, 11.0, 3.0 Hz). An isolated aromatic proton at δ 6.56 (1H, s) and three protons in an ABC system (δ 6.36 (1H, d, *J* = 3.0 Hz), 6.38 (1H, dd, *J* = 8.0, 3.0 Hz) and 6.92 (1H, d, *J* = 8.0 Hz)) indicate that an aromatic ring is penta-substituted, and another ring is tri-substituted. Characterization of Compound **4** was made by comparing the ^1^H-NMR spectrum to that reported for secundiflorol G, a flavan previously described as isolated from *Sophora secundiflora* [13]. This study is the first report on the presence of chalcones and flavans in the genus *Aeschynomene* and, particularly, on the contribution to the phytochemistry of the species *A. fascicularis*.

### 2.2. Biological Activities

This study was conducted towards the isolation of active compounds from the root bark of *A. fascicularis*. Our guided fractionation study suggests that flavan **4** and chalcones **2** and **3** are the main compounds responsible for the observed biological activity in the original extract and the active hexane fraction. The results of the bioassays towards six human cancer cell lines and their selectivity indices (SI) compared with a normal cell line are presented in Table 2, Table 3, Table 4 and Table 5.

Compound **1** did not show any activity on tested cell lines to the maximum concentration (100 µM).

Compound **2** showed good cytotoxic activity against the DU-145 and PC-3 cell line; moreover, this compound showed high selectivity compared with a normal cell line. Interestingly, only this compound showed good antiproliferative activity toward the KB cell line (Table 4).

Compound **3** showed cytotoxic activities on Hep-2, KB and DU-145 cell lines; however, this compound showed poor selectivity (SI) towards all tested cell lines. Cytotoxic activity of isocordoin (**3**) on ovarian, prostate and leukemia human cancer cell lines has been documented [14,15]. Furthermore, isocordoin has been shown to be active against the parasites *Leishmania mexicana* and *Trypanosoma cruzi* [14]. The present work contributes to showing isocordoin to be cytotoxic against Hep-2, HeLa and SiHa cancer cell lines, in which this molecule had not been tested before.

**Table 2:** Cytotoxic activity (CC_50_) of extract, fractions and compounds isolated from *A. fascicularis* root bark.

Fractions/Compound	Cell Lines CC_50_ µg/mL (µM)						
	MDCK	Hep-2	KB	HeLa	SiHa	DU-145	PC-3
**Crude extract**	150.3 (ND)	55.3 (ND)	14.0 (ND)	16.7 (ND)	24.0 (ND)	23.5 (ND)	50.2 (ND)
**Hexane fraction**	175.4 (ND)	33.7 (ND)	21.5 (ND)	18.9 (ND)	30.1 (ND)	29.1 (ND)	21.6 (ND)
**Dichloromethane fraction**	- ^a^	-	-	-	-	-	-
**Ethyl acetate fraction**	-	-	-	-	-	-	-
**Aqueous fraction**	-	-	-	-	-	-	-
**1**	-	-	-	-	-	-	-
**2**	92.1 (245.0)	-	-	-	-	6.1 (16.3)	9.7 (26.0)
**3**	4.2 (14.0)	6.1 (20.0)	11.0 (27.0)	-	-	7.0 (23.1)	18.4 (60.3)
**4**	29.7 (83.4)	3.9 (11.0)	5.3 (15.0)	8.1 (23.0)	14.7 (41.3)	21.5 (60.6)	16.5 (46.4)
**Docetaxel**	1.1 (1.4)	0.07 (0.1)	0.2 (0.3)	0.19 (0.25)	0.17 (0.22)	0.007 (0.01)	0.07 (0.1)

^a^ >100 µg/mL. ND = not determined; range of activity in µg/mL: <5 = very strong; 5–10 = strong; 10–20 = moderate; 20–100 = weak; >100 = not active [16].

**Table 3:** Selectivity index (SI) of cytotoxic activity of compounds isolated from *A. fascicularis* root bark.

Compound	Cell Lines SI					
	Hep-2	KB	HeLa	SiHa	DU-145	PC-3
**Crude extract**	2.7	10.7	9.0	6.3	6.3	3.0
**Hexane fraction**	5.2	8.1	9.2	5.8	6.0	8.1
**Dichloromethane fraction**	-	-	-	-	-	-
**Ethyl acetate fraction**	-	-	-	-	-	-
**Aqueous fraction**	-	-	-	-	-	-
**1**	- ^a^	-	-	-	-	-
**2**	-	-	-	-	15.0	9.4
**3**	0.7	0.5	-	-	0.6	0.23
**4**	7.6	5.5	3.6	2.0	1.3	1.8
**Docetaxel**	14.0	4.6	5.6	6.3	140	14.0

^a^ Undetermined; not selective when SI < 10 [12].

**Table 4:** Antiproliferative activity (IC_50_) of compounds isolated from *A. fascicularis* root bark.

Compound	Cell Lines IC_50_^a^ µg/mL (µM)						
	MDCK	Hep-2	KB	HeLa	SiHa	DU-145	PC-3
**1**	- ^a^	-	-	-	-	-	-
**2**	18.7 (50.0)	-	6.0 (16.0)	-	-	-	-
**3**	18.4 (60.8)	-	-	-	-	13.3 (43.8)	-
**4**	25.4 (72.0)	-	-	-	21.2 (60.0)	-	-
**Docetaxel**	0.11 (0.14)	0.05 (0.07)	0.04 (0.06)	0.03 (0.04)	0.07 (0.1)	0.01 (0.02)	0.007 (0.01)

^a^ >100 µM; range of activity in µg/mL: <5 = very strong; 5–10 = strong; 10–20 = moderate; 20–100 = weak; >100 = not active [16].

**Table 5:** Selectivity index (SI) of antiproliferative activity of compounds isolated from *A. fascicularis* root bark.

Compound	Cell Lines IC_50_ SI					
	Hep-2	KB	HeLa	SiHa	DU-145	PC-3
**1**	- ^a^	-	-	-	-	-
**2**	-	3.1	-	-	-	-
**3**	-	-	-	-	1.4	-
**4**	-	-	-	1.2	-	-
**Docetaxel**	2.0	2.3	3.5	1.4	7	14

^a^ Undetermined; not selective when SI < 10 [17].

Secundiflorol G (**4**) showed potent activity and moderate selectivity towards oral-laryngeal cancer cell lines Hep-2 and KB. Previously, the cytotoxic activity of various isoflavans had been reported against mouth cancer cell lines [18,19] and is consistent with the results obtained in this work for secundiflorol G (**4**) towards KB and Hep-2 lines. Therefore, we propose the potential use of secundiflorol G in oral-laryngeal cancer chemotherapy.

This is the first report on the pharmacology of Compounds **1**, **2** and **4**. Furthermore, it can be deduced that cytotoxic activity observed for the methanol extract and hexane fraction can be attributed to Compounds **2**, **3** and **4**. The mechanism of action by which chalcones and isoflavans exert their cytotoxic activity remains undiscovered; however, Sabzevari [20] described chalcone-type compounds to have a mechanism of free radical toxicity. In addition to this, it has been elucidated that the main mechanism of action of chalcones is inducing *in vitro* and *in vivo* apoptosis [21,22,23]. By contrast, the mechanism by which isoflavans exert their cytotoxicity has not been well elucidated.

Finally, spinochalcone A (**2**) is the most abundant compound present, as much as 30% of the hexane fraction of *A. fascicularis* root bark (measured by GC-MS), and this could be exploited to develop **2** as a potential marker molecule (together with **3** and **4**) to standardize the hexane fraction of *A. fascicularis* as a phytomedicine. In addition to this, it is worth highlighting the antiproliferative effect that **2** presented against the KB cell line, providing an added value to the development of *A. fascicularis* as a phytomedicine for the treatment of oral-laryngeal cancer. At present, cancer therapies are using multiple cytotoxic drugs acting synergistically, in order to enhance the curative potency of all substances together. This emphasizes the effectiveness of a phytomedicine prepared from the hexane fraction of *A. fascicularis*.

The present study suggests that chalcones **2** and **3** and flavan **4** are the main compounds responsible for the observed biological activity in the original extract of *A. fascicularis* and are potential candidates for drug development based on their effective cytotoxic and antiproliferative activities.

## 3. Experimental Section

### 3.1. General

UV spectra were taken in CH_2_Cl_2_ using a Beckman Coulter DU-650 spectrophotometer (Brea, CA, USA). The FTIR spectra were measured with a Nicolet Protégé spectrophotometer (Madison, WI, USA). NMR spectra (^1^H, ^13^C, DEPT, COSY, HSQC and HMBC) were acquired on a Bruker Avance (Billerica, MA, USA) 400 Ultra Shield spectrometer and TMS was used as an internal standard. Low-resolution mass spectra were taken on an Agilent Technologies (Santa Clara, CA, USA) 6890N gas chromatograph coupled to a 5975B mass spectrometer (GC-MS). Column chromatography was carried out on silica gel 60 (70–230 mesh) from Merck (Darmstadt, Germany) and Sephadex LH-20 from Sigma (St. Louis, MO, USA). TLC was performed on precoated silica gel 60 F_254_ (0.25 mm) (Merck). Spots on TLC were visualized under UV light and by spraying phosphomolybdic acid reagent, followed by heating. The optical density of the developed plates with the 3-(4,5-dimethylthiazol-2-yl)-2,5-diphenyl tetrazolium bromide (MTT, Sigma) and sulforhodamine B (SRB, Sigma) was read in a ThermoSpectronic spectrophotometer from Bio-Rad (Hercules, CA, USA).

### 3.2. Plant Material

Root bark of *A*. *fascicularis* was collected in May 2009 from Yaxcabá, Yucatan (latitude 20°38′06′′N; longitude 88°48′42′′W) (Mexico). The plant material was identified and authenticated by a taxonomist from the Natural Resources Unit of the Centro de Investigación Científica de Yucatán (CICY). Specimens under the voucher number P. Simá 2997 were deposited at CICY’s U Najil Tikin Xiw herbarium.

### 3.3. Isolation of Active Compounds

Dried powdered root bark (594 g) was extracted by maceration at room temperature with MeOH (3 × 500 mL) for 24 h. The supernatants were filtered and evaporated under vacuum by means of a rotary evaporator to obtain a dried extract. The MeOH extract (84 g) was suspended in 500 mL MeOH/H_2_O (1:3) and extracted using solvents of increasing polarity (3 × 1000 mL): hexane, CH_2_Cl_2_ and EtOAc. The solvents were removed using a rotary evaporator (40 °C) to obtain the hexane, dichloromethane, ethyl acetate and aqueous fractions. Only the hexane fraction showed cytotoxic activity against the Hep-2 cancer cell line (CC_50_ = 18 µg/mL).

The active hexane fraction (6.4 g) was submitted to a vacuum liquid chromatography (VLC) on silica gel using mixtures of hexane (Hx) and acetone (An) of ascending polarity to yield 11 fractions, which were pooled into six final fractions (A1–A6), according to their similarity on TLC. Fractions A2 and A3 were each purified by silica gel column chromatography (CC) under isocratic elution with Hx/An (9:1) to yield spinochalcone C (**1**, 60 mg) and spinochalcone A (**2**, 65 mg), respectively. Fraction A4 was explored using a Sephadex LH-20 CC to obtained four fractions (B1–B4). Fraction B2 was eluted with MeOH (100%) and was further subjected to CC silica gel using the isocratic system Hx/An (8:2) to obtain Compound **3** (5 mg). Finally, from Fraction A6, Compound **4** (20 mg) was isolated by means of a CC silica gel using the solvent system CH_2_Cl_2_/MeOH (9:1).

### 3.4. Cell Culture

Three types of cancer cell lines obtained from the American Type Culture Collection (ATCC) were cultured: two oral-laryngeal cancer cell lines, nasopharynx carcinoma (KB, ATCC-CCL-17) and laryngeal carcinoma (Hep-2, ATCC-CCL-23); two cervix-uterine cancer cell lines, cervix adenocarcinoma (HeLa, ATCC-CCL-2) and cervix squamous carcinoma (SiHa, ATCC-HTB-35); and two prostate cancer cell lines, prostate carcinoma (DU-145, ATCC-HTB-81) and prostate adenocarcinoma (PC-3, ATCC-CRL-1435); along with one normal cell line (MDCK, ATCC-CCL-34). The cells were cultured in sterile Costar T75 flasks containing DMEM (Gibco, Hong Kong, China), supplemented with fetal bovine serum (10%, *v*/*v*), 100 U/mL penicillin G, and 100 mg/mL streptomycin at 37 °C in an atmosphere of 5% CO_2_ (95% humidity). At 70%–80% confluence, cells were detached from the cultured flask by treatment with 0.05% trypsin-EDTA (Gibco), and a suspension of 5 × 10^4^ cells/mL of viable cells was seeded in a 96-well microtiter plate (Costar, Washington, DC, USA) and incubated.

### 3.5. Cytotoxicity Assay

Cytotoxicity was assessed by the MTT assay as previously described by Rahman [24]. When cells reached 90% confluence in a microtiter plate, the DMEM was replaced with fresh medium and cells were incubated with compounds at various concentrations. After 72 h of incubation, 10 μL of 3-(4,5-dimethylthiazol-2-yl)-2,5-diphenyl tetrazolium bromide (MTT, Sigma) solution (5 mg/mL) were added to each well and incubated at 37 °C for 4 h. The medium was aspirated, and the formazan product was solubilized with acidified isopropanol (0.4 *N* HCl). The amount of MTT-formazan is directly proportional to the number of living cells and was determined by measuring the optical density (OD) at 590 nm using a bioassay reader (Bio-Rad). All determinations were performed in triplicate.

### 3.6. Antiproliferative Assay

The growth inhibition of cancer cells was evaluated by the sulforhodamine B method [25]. When cells reached 70% confluence in a microtiter plate, the medium was replaced with DMEM 5% FBS and samples at various concentrations. After 48 h, the medium was discarded, and cells were fixed by adding 50 μL of 10% trichloroacetic acid (TCA, Sigma-Aldrich Chemical, St. Louis, MO, USA). The cells were then incubated at 4 °C for 30 min; TCA was drained off, and the plates were left to dry. Then, 50 μL of SRB stain (10 mg of 1% acetic acid, Sigma) were added to each well for 30 min. Finally, the plates were washed four times with 1% acetic acid (100 μL). The OD was measured at 540 nm using an ELISA reader (Bio-Rad). Docetaxel was used as a positive control, whereas cells incubated only with 0.05% of DMSO were used as a negative control. All determinations were performed in triplicate.

### 3.7. Data Analysis

The cytotoxic and antiproliferative activities of tested compounds were calculated as the percentage of cells killed or the percentage of inhibition of cell proliferation, respectively, using the equation: growth inhibition (%) = (OD_control_ − OD_sample_/OD_control_) × 100. Results are expressed as the concentration of compound that killed 50% of the cells (CC_50_) and that reduced cell growth by 50% (IC_50_), respectively, and they were calculated using the software GraphPad Prism 4. In addition, the level of cytotoxicity on normal cells was evaluated by determining the selectivity index (SI) of each compound, which is calculated as the ratio of cytotoxicity or reduced cell growth on normal cells to cancer cells (SI = normal cells/cancer cells) [26]. It is generally considered that biological efficacy is not due to general toxicity when SI ≥ 10 [17].

## 4. Conclusions

The present study suggests that chalcones **2** and **3** and flavan **4** are the main compounds responsible for the observed biological activity in the original extract of *A. fascicularis* and are potential candidates for drug development based on their effective cytotoxic and antiproliferative activities.

## Abbreviations

λ_max_: wavelength of maximum absorption
ν_max_: frequency of the maximum height in the infrared spectrum
a.m.u.: atomic mass unit
COSY: correlation spectroscopy
DMSO: dimethyl sulfoxide
DEPT: distortionless enhancement by polarization transfer
EI-MS: electronic impact mass spectrum
FTIR: Fourier transform infrared spectroscopy
HSQC: heteronuclear single quantum coherence
HMBC: heteronuclear multiple bond correlation
NMR: nuclear magnetic resonance
OD: optic density
TLC: thin-layer chromatography
